# Editorial: Cell cycle control as a new therapeutic approach for SARS-CoV-2 infection

**DOI:** 10.3389/fphar.2023.1136277

**Published:** 2023-02-01

**Authors:** Roberta Rizzo, Francesca Caccuri, Giuseppe Valacchi, Giorgio Zauli

**Affiliations:** ^1^ Department of Chemical, Pharmaceutical and Agricultural Sciences, University of Ferrara, Ferrara, Italy; ^2^ Department of Molecular and Translational Medicine, University of Brescia, Brescia, Italy; ^3^ Plants for Human Health Institute, North Carolina State University, Kannapolis, NC, United States; ^4^ Research Department, King Khaled Eye Specialist Hospital, Riyadh, Saudi Arabia

**Keywords:** cell cycle, SARS-CoV-2, p53, cytokine storm, idasanutlin

SARS-CoV-2 can manipulate cellular pathways, changing how well they can resist viral infection. Because of SARS-CoV-2 capacity to destroy p53, less cell death occurs in infected cells, which promotes viral replication, and p53 antiviral action is lost. Regarding this, p53 is a pleiotropic molecule associated with antiviral innate immune responses, which are specifically carried out by triggering apoptosis of infected cells and facilitating type I interferon (IFN) production/signaling. p53 consequently plays a crucial role in the setting of antiviral immunity, which may be why it is frequently targeted by viruses. By preventing virus-targeted cells from undergoing apoptosis, inflammation is made worse and a “cytokine storm” is produced.

The major goals of the current pharmacological techniques to control SARS-CoV-2 infection are to prevent viral binding and entry into human cells, to obstruct polyprotein complex translation and proteolysis, to obstruct viral RNA replication, and to limit viral release. The severity of COVID-19 disease is linked to heightened inflammatory responses, according to newly available clinical data, indicating that patient treatment plans should go beyond antiviral drugs.

To contrast SARS-CoV-2 infection and to modulate host immunity and inflammatory responses, this Research Topic explored and covered the current knowledge and research trends of pharmacological treatment for COVID-19 patients that is not targeted against SAS-CoV-2 but on cell cycle control. Two original research articles, one review, and one perspective are presented.

Extracellular vesicles (EVs) are discussed in a perspective paper by Bortot et al. on their potential to control inflammation and cell death in disorders linked to COVID-19. EVs can be used as natural drug delivery nanoparticle-based systems because of their natural origin, innate capacity for material transfer between cells, and ability to encapsulate a variety of biological compounds. The authors propose using EVs to transport small-molecule MDM2 inhibitors like Nutlin-3 and idasanutlin by enhancing p53 survival. Given the possible antiviral and anti-inflammatory effects, this strategy is promising. It may help modify the IFN signaling pathway and lessen the overall pro-inflammatory burden during COVID-19.

To control viral replication in host cells, Milani et al. provided a review of various molecular pathways by which SARS-CoV-2 modifies p53 and NF-kB expression and activity. As a result of SARS-CoV-2 infection, important molecules such as the RAS pathway member ACE2, the virus–host cell entrance mediator p53, and the transcriptional regulator NFkB are all changed. These modifications are believed to be the primary cause of the compromised immune response and the large cytokine release, which is a defining feature of the most severe SARS-CoV-2 infection. Following the idea of precision and tailored medicine, the authors suggest multi-OMICs and pharmacogenomics investigations to characterize the wide range of clinical manifestations of COVID-19 and establish as many therapeutic options as possible to be able to control disease symptoms when vaccination does not offer enough protection or is not feasible. Considering the widely acknowledged important function of pathways’ mutual crosstalk in inflammatory conditions, reestablishing the proper balance between p53 and NF-kB is a possible therapeutic strategy. To this end, the development of new active compounds and the repositioning of existing medications (as suggested for idasanutlin) may be of great significance in the context of synergistic treatments capable from one side of improving an individual’s immune status and from the other side of targeting pathways involved in the development and progression of COVID-19.

Considering the rising number of non-small-cell lung cancer (NSCLC) patients who also have COVID-19, Zhuang et al. suggested evaluating the effect of COVID-19 on lung cancer patients. Twenty-one interactional hub genes and a total of 122 COVID-19/NSCLC interactional genes were evaluated. The enrichment analysis revealed that the signaling pathways involved in cell cycle, viral carcinogenesis, and p53 signaling were shared by COVID-19 and NSCLC. In total, 21 interactional hub genes were regulated by 44 microRNAs and 10 significant transcription factors (TFs). Additionally, 23 viable candidates were anticipated for the management of COVID-19 and NSCLC. These findings confirm the necessity to find new COVID-19 and NSCLC medication candidates that may work on identified shared pathways.

In their original study, Lodi et al. explored a novel strategy for overcoming SARS-CoV-2 infection by employing MDM2 inhibitors to increase p53 levels and activate p53-dependent pathways, which inhibits cell division. The alveolar basal epithelial cell line A549-hACE2 was used as the experimental subject because it expresses high levels of the ACE2 receptor, which facilitates virus entrance, and the wild-type p53. For the establishment of a cell cycle block steady-state condition prior to and during SARS-CoV-2 infection, as well as for the evaluation of p53 activation and its impact on virus release and related innate immune events, cells were treated with various concentrations of Nutlin-3 or RG-7112, two known MDM2 inhibitors. The acquired data demonstrated an effective cell cycle block together with a considerable inhibition of IL-6, NF-kB, and IFN expression. These findings point to p53 as an effective target for novel treatments to control SARS-CoV-2 infection.

The Guest Editors would like to express their gratitude to all the authors and reviewers for their efforts and dedication to the Research Topic and express the hope it can inspire additional studies into the treatment of SARS-CoV-2 infection.

